# Enhancing Immune Response in Non-Small-Cell Lung Cancer Patients: Impact of the 13-Valent Pneumococcal Conjugate Vaccine

**DOI:** 10.3390/jcm13051520

**Published:** 2024-03-06

**Authors:** Jolanta Smok-Kalwat, Paulina Mertowska, Izabela Korona-Głowniak, Sebastian Mertowski, Paulina Niedźwiedzka-Rystwej, Dominika Bębnowska, Krzysztof Gosik, Andrzej Stepulak, Stanisław Góźdź, Jacek Roliński, Zofia Górecka, Jan Siwiec, Ewelina Grywalska

**Affiliations:** 1 Department of Clinical Oncology, Holy Cross Cancer Centre, 3 Artwinskiego Street, 25-734 Kielce, Poland; jolantasm@onkol.kielce.pl (J.S.-K.); stanislawgo@onkol.kielce.pl (S.G.); 2 Department of Experimental Immunology, Medical University of Lublin, 4a Chodzki Street, 20-093 Lublin, Poland; sebastian.mertowski@umlub.pl (S.M.); krzysztof.gosik@umlub.pl (K.G.); ewelina.grywalska@umlub.pl (E.G.); 3 Department of Pharmaceutical Microbiology, Medical University of Lublin, 1 Chodzki Street, 20-093 Lublin, Poland; iza.glowniak@umlub.pl; 4 Institute of Biology, University of Szczecin, Felczaka 3c, 71-412 Szczecin, Poland; paulina.niedzwiedzka-rystwej@usz.edu.pl (P.N.-R.); dominika.bebnowska@usz.edu.pl (D.B.); 5 Department of Biochemistry and Molecular Biology, Medical University of Lublin, 1 Chodzki Street, 20-093 Lublin, Poland; andrzej.stepulak@umlub.pl; 6 Institute of Medical Science, Collegium Medicum, Jan Kochanowski University of Kielce, IX Wieków Kielc 19A, 25-317 Kielce, Poland; 7 Department of Clinical Immunology, Medical University of Lublin, 4a Chodzki Street, 20-093 Lublin, Poland; jacek.rolinski@umlub.pl; 8 Department of Plastic and Reconstructive Surgery and Microsurgery, Medical University of Lublin, 8 Jaczewskiego Street, 20-090 Lublin, Poland; zfgrecka@gmail.com; 9 Department of Pneumonology, Oncology and Allergology, Medical University of Lublin, 8 Jaczewskiego Street, 20-090 Lublin, Poland; jan.siwiec@umlub.pl

**Keywords:** non-small-cell lung cancer, immune system, 13-valent pneumococcal conjugate vaccine, *Streptococcus pneumoniae*, vaccination

## Abstract

**Background:** Non-small-cell lung cancer (NSCLC) is one of the most frequently diagnosed diseases among all types of lung cancer. Infectious diseases contribute to morbidity and mortality by delaying appropriate anti-cancer therapy in patients with NSCLC. **Methods:** The study aimed to evaluate the effectiveness of vaccination with the 13-valent pneumococcal conjugate vaccine (PCV13) in 288 newly diagnosed NSCLC patients. The analysis of the post-vaccination response was performed after vaccination by assessing the frequency of plasmablasts via flow cytometry and by assessing the concentration of specific anti-pneumococcal antibodies using enzyme-linked immunosorbent assays. **Results:** The results of the study showed that NSCLC patients responded to the vaccine with an increase in the frequencies of plasmablasts and antibodies but to a lesser extent than healthy controls. The immune system response to PCV13 vaccination was better in patients with lower-stage NSCLC. We found higher antibody levels after vaccination in NSCLC patients who survived 5 years of follow-up. **Conclusions:** We hope that our research will contribute to increasing patients′ and physicians′ awareness of the importance of including PCV13 vaccinations in the standard of oncological care, which will extend the survival time of patients and improve their quality of life.

## 1. Introduction

Non-small-cell lung cancer (NSCLC) is one of the most commonly diagnosed entities of lung cancer [1,2]. Numerous interdisciplinary studies are currently investigating factors contributing to the development and progression of this particular form of cancer, including the influence of environmental factors, genetics, and, significantly, the immune system’s involvement in its pathogenesis [3,4,5]. The involvement of microorganisms in pathogenesis, particularly changes in the lung microbiome, and the development of infections were widely reported [6,7,8,9,10,11]. Infections may be one of the most common causes of lung cancer progression, associated with decreased patient response to treatment, poorer prognosis, and increased mortality [10,11]. One of the newer research areas observed in recent years is the discussion of the role and involvement of *Streptococcus pneumoniae* (not only in the progression of lung cancer but also in its participation in the process of carcinogenesis itself) [12,13,14,15]. This is related to the increased susceptibility to infections caused by these Gram-positive bacteria, which can cause invasive and non-invasive pneumococcal disease, observed among cancer patients. Infection caused by *S. pneumoniae* may promote the pathomechanism of the carcinogenesis process by increasing the proliferation and migration of neoplastic cells in the course of NSCLC, and their increased number observed in studies correlates with the development of cancer [12,13,14]. The increased incidence of pneumococcal infections among NSCLC patients is an extremely difficult therapeutic challenge and significantly affects the survival rates of cancer patients [16,17,18]. Database analysis showed that out of 105,060 articles on NSCLC, only 691 deal with the occurrence of pneumonia, and only 3 highlight the importance of vaccination. To protect oncology patients, especially those with NSCLC, from the harmful effects of *S. pneumoniae* infections, it is essential to use preventive vaccinations. Previous studies performed with the 23-valent pneumococcal polysaccharide vaccine (PPSV23) demonstrated that the immunogenicity of pneumococcal vaccines in cancer patients could induce adequate immune responses in patients undergoing chemotherapy [19,20]. Moreover, a recent study reported that the 13-valent pneumococcal conjugate vaccine (PCV13) is safe and immunogenic in children who completed cancer treatment [21]. However, data on PCV13 use in adult cancer patients are still scarce.

This study aimed to assess the post-vaccination response of patients diagnosed with NSCLC after administration of the PCV13 vaccine concerning their survival rate and cancer stage. Our understanding of vaccine response will be crucial in introducing a successful, customized approach to infection prevention, referring to leading complications in these patients and potentially decreasing associated morbidity and mortality.

## 2. Materials and Methods

### 2.1. Characteristics of the Patients with NSCLC Included in the Study and the Control Group

**Figure 1:**
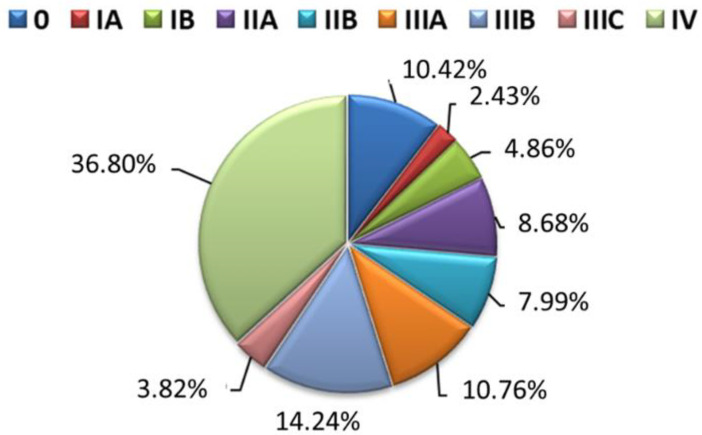
Tumor staging of NSCLC patients at admission based on the TNM scale.

The study included 288 patients from the Świętokrzyskie province (Poland) diagnosed with NSCLC at various stages of cancer (Figure 1). Patients were admitted for treatment to the Holycross Cancer Center in Kielce (Poland) in the years 2014–2015. The mean age of the patients in the study group was 52.36 ± 9.12 (Table 1). This study focused on patients diagnosed with NSCLC who had not previously received any treatment for the disease, including chemotherapy and immunotherapy. Blood samples were taken from these patients to analyze NSCLC grade and stage. Certain exclusion criteria were established to ensure accurate results, including prior vaccination against *S. pneumoniae*, medication affecting the immune system, recent infection, blood transfusion history, autoimmune disease, cancer, allergies, and pregnancy or lactation within the past year. The patients had not been diagnosed with other diseases or were taking any medications that could affect the results of the study before and 30 days after vaccination. Anti-cancer treatment was initiated in all patients 30 days after the vaccination, just after the blood was collected for the tests that are the subject of this study. The implemented treatment depended on the cancer stage and included standard methods: surgery, radiotherapy, chemotherapy, molecularly targeted drugs, immunotherapy, and combined methods. There were no differences in post-vaccination response or infection rates depending on the applied anti-cancer therapy. All study participants and a control group of healthy individuals matched in age. None of the study participants had COVID-19 or received any preventive vaccinations other than PCV13 during follow-up. After giving consent, 52.43% of patients were vaccinated against pneumococci. The control group consisted of 69 people, age-matched to the study group (53.47 ± 10.14), who voluntarily underwent pneumococcal vaccination. The control group’s health was verified through regular diagnostic evaluations conducted during visits to a general practitioner. Among the control group, 49.27% of the patients recruited for the study received a protective vaccine against pneumococcus.

### 2.2. Research Material

For each patient in the control and study groups, we collected peripheral blood (PB) from the basilic vein at three different times: before vaccination, 7 days after vaccination, and 30 days after. The collected blood was used to perform the following tests: (1) measure the level of specific anti-pneumococcal antibodies in the serum before and 30 days after vaccination (3 mL of PB was collected into coagulation factor tubes); (2) determine the percentage of plasmablasts, identified as CD19+/IgD2/CD27++, at three specific time intervals (5 mL of PB collected in tubes with EDTA anticoagulant); and (3) concentration of IgG in serum and IgG1, IgG2, IgG3, and IgG4 at three time points (5 mL of PB collected in tubes with coagulation activator). In addition, a blood count was performed each time with an additional biochemical determination of C-reactive protein (CRP). Samples of serum were kept at −80 °C until analysis of the level of specific anti-pneumococcal antibodies. We assessed the percentage of plasmablasts in fresh PB samples from both NSCLC patients and healthy volunteers. Additionally, the level of IgG and its subclasses was measured in fresh serum samples. We conducted further examinations of patients in the study group at 1, 2, 3, 4, and 5 years after vaccination or diagnosis of NSCLC to monitor changes in post-vaccination response over time.

### 2.3. Vaccine

Patients diagnosed with NSCLC and a control group received a 13-valent subunit conjugate vaccine (PCV13) called Prevenar13, made by Pfizer. The vaccine contains polysaccharide antigens from various pneumococcal serotypes, including 1, 3, 4, 5, 6A, 6B, 7F, 9V, 14, 18C, 19A, 19F, and 23F. The vaccine was given to all patients once through an intramuscular injection according to the manufacturer’s instructions. None of the patients in either group had received a pneumococcal vaccine before.

### 2.4. Plasmablasts Evaluation

To prepare for testing, the blood samples were mixed with a solution of phosphate-buffered saline that contained no calcium or magnesium. This mixture was then layered onto a substance called Gradisol L (Aqua Medica, Poznań, Poland) and spun in a centrifuge for 20 min. The resulting cells (PBMC) were collected and washed twice with the same calcium and magnesium-free solution. After that, the cells were counted, and their vitality was checked using trypan blue (0.4% Trypan Blue Solution, Sigma Aldrich, Darmstadt, Germany). To test the response to the 13-valent pneumococcal conjugate vaccine in patients, PBMCs were separated using density gradient centrifugation and labeled with monoclonal antibodies according to the manufacturer’s instructions. Each sample was incubated with 20 µL of antibodies at room temperature for 20 min before being washed twice with PBS and analyzed using the FACSCalibur flow cytometer. We used the FACS Diva Software 6.1.3 system in the data acquisition process and the CellQuest Pro software for data analysis, both from Becton Dickinson. To exclude erythrocytes, platelets, dead cells, and cell fragments from analysis, 30,000 events were collected in the lymphocyte gate in a forward-scatter (FSC)/side-scatter (SSC) dot plot for each sample. Labeled cells were recorded based on the created lymphocyte gate, and the results were presented as a percentage of CD45+ cells stained with the antibody. To conduct our study, we utilized a variety of monoclonal antibodies that were conjugated with appropriate fluorochromes. These included FITC-mouse anti-human IgM, FITC-mouse anti-human IgD, PE-mouse anti-human CD19, PE-mouse anti-human CD38, PE-Cy5-mouse anti-human CD19, and APC-mouse anti-human CD27 (all from Becton Dickinson, Holdrege, NE, USA). To ensure accuracy, we also utilized mouse isotype controls, including FITC Mouse IgG1k, Isotype Control, Clone MOPC-21, FITC Mouse IgG2a k, Isotype Control, Clone G155-178, PE Mouse IgG1 k, Isotype Control, Clone MOPC-21, and APC Mouse IgG1k, Isotype Control, respectively. We evaluated the proportion of plasmablasts in the peripheral blood on the day of vaccination and 7 and 30 days following vaccination.

### 2.5. Serum Pneumococcal Antibody Assessment

Before and after vaccination, all subjects underwent a serum pneumococcal antibody assessment. This involved measuring the amount of anti-capsular-polysaccharides antibody specific for 23 different pneumococcal serotypes (1, 2, 3, 4, 5, 6B, 7F, 8, 9N, 9V, 10A, 11A, 12F, 14, 15B, 17F, 18C, 19A, 19F, 20, 22F, 23F, and 33F) using a commercial ELISA test (ELIZEN Pneumococcus IgG Assay, Zentech, Liège, Belgium). To increase the test’s specificity, each serum sample was pre-adsorbed with 10 mg/mL polysaccharide C (C-PS, Statens Serum Institute, København, Denmark) for 1 h at 37 °C. The manufacturer’s instructions were followed during the evaluation process, and a VICTOR3 reader (Perkin Elmer, Waltham, MA, USA) was used for result interpretation.

### 2.6. Assessment of IgG Subclasses

The IgG subclasses (IgG1, IgG2, IgG3, and IgG4) were evaluated using a nephelometer BN2 (Dade Behring, Marburg, Germany) through nephelometric techniques. The process was carried out following the instructions provided by the manufacturer.

### 2.7. Statistical Analysis

Statistical information about continuous variables was presented, including the median, minimum, and maximum values, arithmetic means, and standard deviations (SD). We used methods such as the Mann–Whitney U test and Spearman rank order correlations to compare between groups. Logistic regression models were fitted to identify factors associated with NSCLC patient’s survival. Separate multivariate models were constructed for three periods of time (before vaccination, 7 days, and 30 days after vaccination). The variance inflation factor (VIF) was calculated to estimate a multicollinearity of each predictor with all the other predictors. A backward elimination model including blood parameters examined in respective time periods was built, and nonsignificant variables were removed sequentially until only those significant at *p* < 0.1 remained. From these models, adjusted odds ratios (OR) and 95% confidence intervals were derived; corresponding *p*- values were from Wald’s test. The goodness-of-fit was checked using Hosmer and Lemeshow’s test. We performed all calculations using Statistica 13 (StatSoft, Tulsa, OK, USA) and considered a significance level of *p* < 0.05.

## 3. Results

### 3.1. Evaluation of Post-Vaccination Response to PCV13 in NSCLC Patients and Controls

When patients were recruited for the NSCLC and the control groups, an analysis of selected parameters of PB counts and the level of the C-reactive protein (CRP) was performed. The obtained test results are presented in Table 2. Patients from the study group had higher levels of WBC (1.58 times), MON (1.65 times), NEU (2.04 times), and PLT (1.24 times) than the patients in the control group. Additionally, patients with NSCLC had CRP values more than 12 times higher than those of healthy volunteers. Moreover, these patients also had reduced levels of RBC, hemoglobin, and hematocrit. Considering the heterogeneity of the NSCLC group, the differences in blood parameters among patients in the early stages of the disease—0 to II stage—and patients in late stages—III and IV—were established (Table 2). The tested blood parameters showed no significant differences among patients in these groups, but the WBC and neutrophil accounts were significantly higher in patients with advanced NSCLC (*p* = 0.002 and *p* = 0.012, respectively).

**Figure 2:**
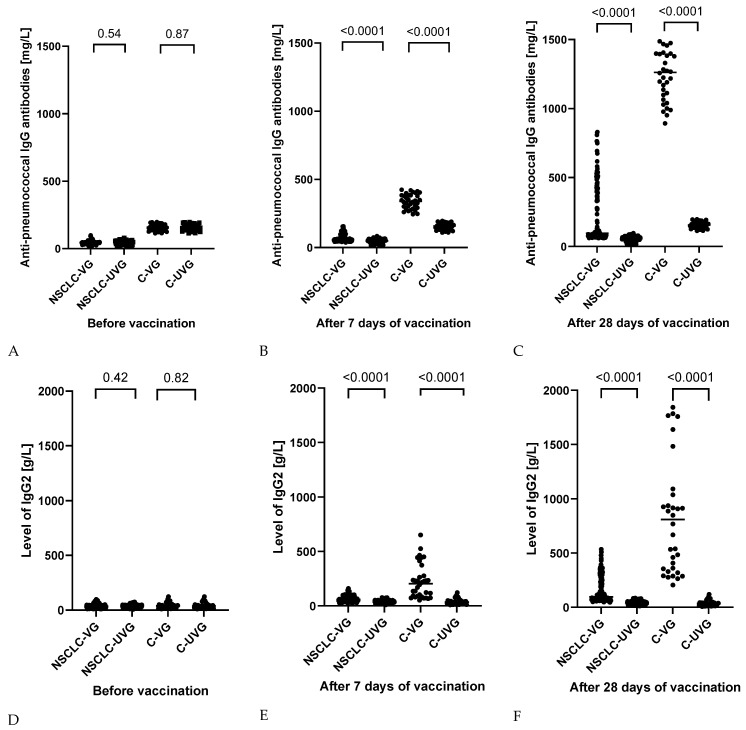
Changes in the level of anti-pneumococcal IgG antibodies (**A**–**C**) and the level of individual IgG2 subclass (**D**–**F**) before and after 7 and 30-day periods from the moment of vaccination (NSCLC-VG—NSCLC vaccinated group, NSCLC-UVG—NSCLC unvaccinated group, C-VG—control vaccinated group, and C-UVG—control unvaccinated group).

**Figure 3:**
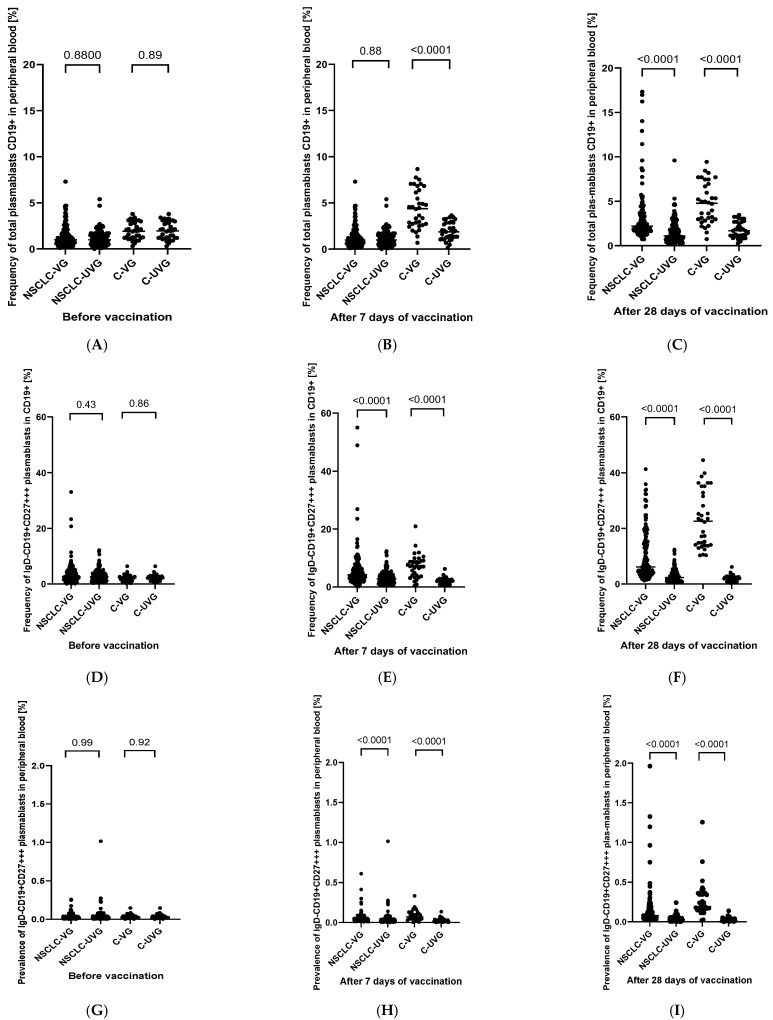
Frequency of total plasmablasts CD19+ in peripheral blood (**A**–**C**); frequency of IgD-CD19+CD27+++ plasmablasts in CD19+ (**D**–**F**) and prevalence of IgD-CD19+CD27+++ plasmablasts in peripheral blood (**G**–**I**) in vaccinated versus non-vaccinated patients in both groups tested before and after 7 and 30-days periods from the moment of vaccination. (NSCLC-VG—NSCLC vaccinated group, NSCLC-UVG—NSCLC unvaccinated group, C-VG—control vaccinated group, and C-UVG—control unvaccinated group).

Of the 288 patients diagnosed with NSCLC, 151 patients were voluntarily vaccinated with PCV13 (52.43%), and 34 (49.27%) were in the control group. To evaluate the response to the vaccine, we analyzed the levels of anti-pneumococcal IgG antibodies and subclasses of IgG antibodies, as well as the percentage of plasmablasts (IgD-CD19+CD27+++) before vaccination, 7 days after vaccination, and 30 days after vaccination. The test results are chronologically presented in Table 3, Figure 2 and Figure 3.

NSCLC patients already had significantly lower levels of anti-pneumococcal antibodies at the time of recruitment compared to healthy volunteers (Figure 2). In addition, we observed that patients from the NSCLC group were also characterized by lower levels of IgG1, IgG2, and IgG3 subclasses, selective deficiency associated with susceptibility to viral and bacterial infections, and especially capsulated bacteria, which include *S. pneumoniae*. According to the data reported in the other studies, a decrease in the value of IgG2 and IgG3 is observed in patients with recurrent infections of the upper and lower respiratory tract [22,23] (Table 3). Changes in the level of anti-pneumococcal antibodies and the level of individual IgG subclasses were checked after 7 and 30 days from the moment of vaccination (Table 3). At the first time point, the antibody level increased by 25.04% for vaccinated NSCLC patients and was 18.02% higher than in the case of unvaccinated patients, for whom there was a slight fluctuation in antibody values. Despite the increase in the level of anti-pneumococcal antibodies in NSCLC patients, the recorded values were significantly lower than the post-vaccination response of healthy volunteers. There was a more than 2-fold increase in antibody levels, which was 4.71-fold higher than in vaccinated lung cancer patients (Figure 2). All observed differences between individual groups of patients were statistically significant.

At the second time point, i.e., 30 days after vaccination, the level of anti-pneumococcal antibodies in vaccinated patients from the NSCLC group was, on average, 4.92 times higher than before vaccination and 3.17 times higher than after 7 days. The observed values were more than 4-fold higher than in unvaccinated lung cancer patients (Figure 2). In the case of patients in the control group, the mean anti-pneumococcal antibody level 30 days after vaccination increased by nearly 8-fold compared to the values recorded before vaccination and 3.69-fold compared to the values after 7 days (Figure 2).

In addition, the level of individual IgG subclasses, which protect the body against bacterial infections, was also analyzed. For both groups of patients who received the vaccination, IgG2 and IgG3 values increased both on the 7^th^ and 30^th^ days after vaccination. In the case of NSCLC patients, the increase in mean IgG2 and IgG3 levels 30 days after vaccination was 1.59-fold and 1.65-fold, respectively. Despite the increase in their levels, the observed values were more than 1.5 times (for IgG2) and 2 times (for IgG3) lower than in the case of healthy subjects (Table 3).

The observed trends of changes in the level of antibodies in individual groups of patients were also reflected in the percentage of peripheral blood plasmablasts. The increase in CD19+ and IgD-CD19+CD27+++ plasmablasts (both CD19+ and total) was significantly higher in vaccinated patients in comparison to the non-vaccinated in both groups tested (Figure 3). Moreover, the rise in plasmablasts was higher in all analyzed cases of healthy patients than in patients diagnosed with NSCLC, which may indicate defects in the functioning of the immune system and show plasmablasts impact on the formation of a normal post-vaccination response (Figure 3). For a broader perspective, Table S1 presents the changes in selected parameters of peripheral blood and CRP levels in NSCLC patients and healthy volunteers in the process of time after vaccination.

Moreover, the point was to estimate if there was a difference in immunological response between patients with early stages of NSCLC and advanced NSCLC (III-IV stages). It turned out that patients from the first group had significantly higher levels of anti-pneumococcal IgG 7 and 30 days after vaccination (Table 4), as well as that the tested plasmablasts revealed significantly higher frequency in cases of patients in early NSCLC at least 30 days after vaccination. Additionally, Table S2 presents the changes in selected parameters of peripheral blood and CRP levels in NSCLC patients divided into two stages of disease groups after vaccination.

### 3.2. Monitoring the Number of Respiratory Infections and Survival Stage of NSCLC Patients after Receiving PCV13 Vaccine

**Figure 4:**
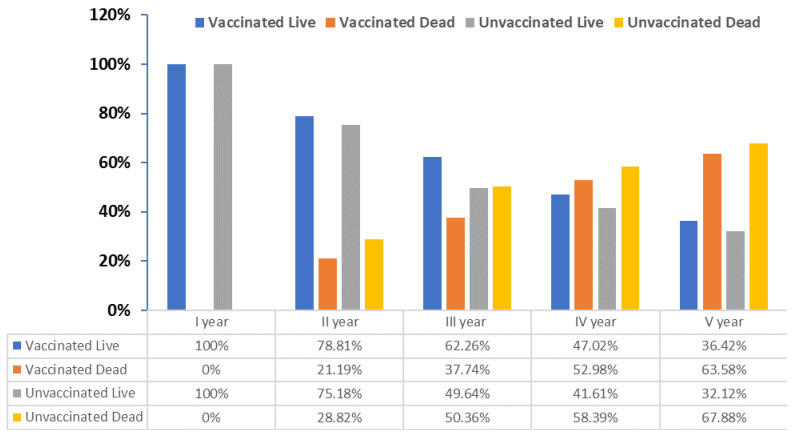
Changes in the number of patients diagnosed with NSCLC over the 5-year study period.

Due to the commencement of treatment at the Holycross Cancer Center, patients diagnosed with NSCLC included in this study were monitored for the number of upper and lower respiratory tract infections caused by *S. pneumoniae* during 5 consecutive years. Unfortunately, over time, 65.62% of patients recruited for the study did not survive the assumed 5-year period. Figure 4 shows the change in the number of patients analyzed each year. All recorded deaths concerned patients whose cancer was in stage III or IV at the time of recruitment for the study. Irrespective of the adoption of the protective anti-pneumococcal vaccination and the applied treatment, these patients could not be saved.

When analyzing the count of upper and lower respiratory tract infections caused by various infectious agents in patients who were vaccinated and unvaccinated with the PCV13 vaccine, we observed that among vaccinated patients with NSCLC, the number of infections per year did not exceed two throughout the study period, while in the case of unvaccinated patients, the number of infections ranged between three and six per year. Detailed analysis showed that among unvaccinated NSCLC patients, the rate of upper and lower respiratory tract infections increased over time elapsed since NSCLC diagnosis, being 23.18% after the first year, 30.85% in the second year, 45.07% in the third year, and reaching 58.18% in the fifth year.

**Figure 5:**
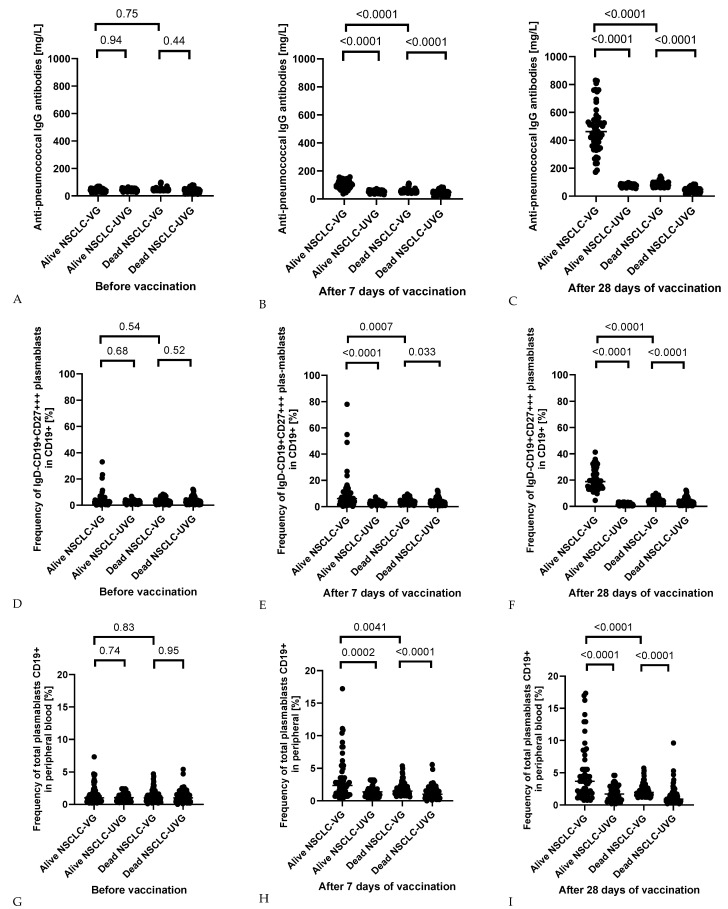
Evaluation of the post-vaccination response of NSCLC patients after receiving PCV13 and unvaccinated NSCLC patients by their survival before vaccination and at the 7th and 30th days after vaccination. Level of anti-pneumococcal IgG antibodies (**A**–**C**); frequency of IgD-CD19+CD27+++ plasmablasts in CD19+ (**D**–**F**) and frequency of total plasmablasts CD19+ in peripheral blood (**G**–**I**). (NSCLC-VG—NSCLC vaccinated group and NSCLC-UVG—NSCLC unvaccinated group).

An additional aspect of our research was to follow up on the post-vaccination response of NSCLC patients every year for 5 years. The results obtained are presented in Table 5. The breakdown of patients with NSCLC, both PCV13-vaccinated and unvaccinated, was received, together with the survival status of the patients. Live vaccinated patients had a significantly higher post-vaccination response than the vaccinated patients who did not survive, who preceded non-vaccinated patients. In the process of time, both the level of anti-pneumococcal antibodies and the percentage of peripheral blood plasmablasts gradually decreased in all groups of analyzed patients. The differences in the potency of response were observed in the level of anti-pneumococcal IgG antibodies, frequency of total plasmablasts CD19+ in peripheral blood, and the frequency of IgD-CD19+CD27+++ plasmablasts in CD19+ after 7 and 30 days after vaccination yet (Figure 5). This observation would be important as a survival prediction factor.

The vast majority of patients who survived the follow-up period were vaccinated and diagnosed at stage 0 or IIA or IIB (RR 1.7, 95%CI 1.3–2.2, *p* < 0.0001), while the patients who died were predominately grade IV (both vaccinated and non-vaccinated, RR 1.1, 95%CI 0.5–2.5, *p* = 0.82). Moreover, multivariate analyses of the association between various clinical characteristics and overall survival were carried out in three periods of time, namely before and at the 7th and 30th days after vaccination. It showed that in the model built for parameters examined 7 days after vaccination, other than an improvement of selected parameters of peripheral blood in time after vaccination, lower NSCLC staging was a statistically significant factor associated with patients’ survival (Table 6). In the multivariate analysis at 30 days after the vaccination period, we observed that patients with lower monocyte and lymphocyte levels but higher hemoglobin, platelet, and leukocyte levels and higher anti-pneumococcal IgG antibody levels had better survival rates.

Due to the recorded percentage of increased mortality in patients with lung cancer diagnosed in stages III and IV, we decided to analyze whether the post-vaccination response of NSCLC patients can correlate with the stage of cancer. The obtained results are presented in Table 5. The post-vaccination response of NSCLC patients shows a significant negative correlation between the level of anti-pneumococcal antibodies, the level of IgG2 and IgG3, as well as the percentage of peripheral blood plasmablasts, and the tumor stage (Table 7). The recorded negative correlation means that with the increase in the stage of cancer, the post-vaccination response of the examined patients decreases in each of the analyzed aspects. In addition, we performed a detailed analysis of the correlation between the level of anti-pneumococcal antibodies, the level of IgG2 and IgG3, and the percentage of peripheral blood plasmablasts in the context of patients who survived and died during the study (Table 7). Vaccinated patients diagnosed with NSCLC who survived the 5 years of the study characterized by a high positive correlation between the level of anti-pneumococcal antibodies and the level of IgG2 (R = 0.698) and IgG3 (R = 0.641), as well as the percentage of total CD19+ peripheral blood plasmablasts (R = 0.627) and IgD -CD19+CD27+++ in CD19+ (R = 0.614), which was maintained throughout the study. In the case of unvaccinated patients who survived the experiment period, a significant low positive correlation was noted between the level of anti-pneumococcal antibodies and the percentage of peripheral blood plasmablasts IgD-CD19+CD27+++ in CD19+ (R = 0.243), which was most likely due to the natural immune response of this group of patients. Low positive correlations were also observed in vaccinated patients who did not survive the 5-year follow-up. These relationships were statistically significant for the level of anti-pneumococcal antibodies and the level of IgG2 (R = 0.211), as well as the total percentage of peripheral blood CD19+ plasmablasts (R = 0.237). In contrast, among unvaccinated patients who died during the study period, a negative correlation was found between the level of anti-pneumococcal antibodies and the total percentage of peripheral blood plasmablasts (R = −0.309). In addition, as the concentration of anti-pneumococcal antibodies increased, the annual incidence of upper and lower respiratory tract infections decreased in vaccinated NSCLC patients, which was not observed in unvaccinated patients.

## 4. Discussion

This study is one of the first evaluations of comprehensive immunological responses in the case of NSCLC patients after anti-pneumococcal vaccination in a 5-year follow-up period. Previous studies, such as those by Mohr et al., indicate that among patients with lung cancer, compliance with recommendations regarding pneumococcal vaccinations, as well as other vaccinations, is low [24]. Therefore, in this context, the analyses conducted by our team, focusing on the immune response, enable arguments about the benefits resulting from the use of protective vaccinations and their impact on patients with NSCLC. The PCV13 vaccine is a preparation widely distributed within many healthcare systems in Poland and around the world. According to the manufacturer’s information, each vaccine dose contains pneumococcal polysaccharides of serotypes 1, 3, 4, 5, 6A, 6B, 7F, 9V, 14, 18C, 19A, 19F, and 23F, which, according to epidemiological data, corresponds to coverage of 50–76% of all cases of invasive pneumococcal disease in adults [25]. Oncological patients are a special group of patients who very often suffer from pneumonia requiring hospitalization, caused not only by *S. pneumoniae* but also by infection with *Staphylococcus aureus* or *Haemophilus influenzae* [11,26]. All these pathogens are characterized by high resistance to commonly used antibiotics and contribute not only to a significant burden on the body’s wealth but also to increased mortality of patients [12,27].

Bacteraemia caused by *S. pneumoniae* in patients with lung cancer can occur in more than 60% of cases [15]. Pneumonia is one of the most common complications diagnosed among cancer patients, especially those with lung cancer. Our findings concerning high levels of monocytes in patients with high risk of NSCLC-associated mortality are similar to those described by Mao et al. [28], who found, among others, higher infiltration of monocytes in the high-risk group of lung squamous cell carcinoma. Moreover, according to Ke et al. [29], high amounts of monocytes were associated with a significantly higher risk of all-cause mortality in asthma patients. It was also proven by Georgakis et al. [30] that the higher circulating monocyte chemoattractant protein-1 (MCP-1) levels were associated with higher long-term cardiovascular mortality in community-dwelling individuals free of overt cardiovascular disease.

The conducted research showed a significant increase in the percentage of both plasmablasts and specific anti-pneumococcal antibodies in voluntarily vaccinated NSCLC patients compared to unvaccinated patients. However, the post-vaccination response of NSCLC patients was significantly lower than that of healthy volunteers who were enrolled as controls, demonstrating the immune burden of NSCLC. Due to the length of the follow-up and the high mortality rate, our study also showed that patients vaccinated with NSCLC who survived for 5 years had significantly higher post-vaccination responses than patients who died, as well as reduced rates of respiratory infections, which may indicate the protective function of the vaccination used. Chiou et al. [31] noted that for elderly lung cancer patients aged ≥75 years, the 23-valent polysaccharide pneumococcal vaccine (PPSV23) inoculated during an anti-cancer treatment period could reduce community-acquired pneumonia hospitalizations and improve survival.

The International Agency for Research on Cancer (IARC) states that lung cancer remains the leading cause of cancer death, with an estimated 1.8 million deaths (18%) in 2020 [32]. In the case of our NSCLC patients, a total of 65.6% died during the 5-year follow-up. Even though there were significant differences in patients’ immune responses about survival states, such differences were not significant about vaccination reception (*p* = 0.46, RR 1.1, 95%CI 0.87–1.37).

Data from the lung cancer report [33] published in 2021, presented by Polish experts, show that by 2040, the ratio of morbidity to mortality in lung cancer patients will reverse, which is associated with the implementation of effective treatment, including molecularly targeted treatment and personalized immunotherapy within the first line of treatment. However, even the best and most advanced therapies will have no chance of success if there are recurrent and severe *S. pneumoniae* infections among cancer patients. Both global and national public health institutions recommend the use of the PCV13 vaccine for specific groups of patients (age > 65 years and suffering from chronic heart and lung diseases, diabetes, alcoholism, nephrotic syndrome, congenital or acquired immunodeficiency, asplenia or post-splenectomy or Hodgkin’s lymphoma), which was proven important based on results presented in this publication, to include oncological patients in said recommendations, especially those diagnosed with lung cancer. Considering that anti-cancer treatment (based on chemo- and/or radiotherapy) significantly affects the functioning of the patient’s immune system, and the aggravating state of its dysfunction contributes to the increased incidence of oncological patients for life-threatening infections, both local (lung infections bronchitis) and generalized (bacteremia). As a consequence of difficult diagnostics or multi-resistance to antibiotics, such infections may, in many cases, lead to premature death of patients [34,35,36].

Potential synergistic effects of PCV vaccination in combination with other immunotherapies or treatments used in NSCLC should also be investigated. The case report by Huang et al. [37] reported that radiotherapy in combination with PCV could specifically stimulate immune response and remodel the tumor–immune microenvironment in tyrosine kinase inhibitor-resistant NSCLC, which may provide a new perspective for future immunotherapy in this challenging clinical situation. Combinations may enhance the overall immune response and lead to better clinical outcomes. It also seems important to develop and validate biomarkers to predict the response of NSCLC patients to PCV vaccination. Immune system monitoring can help to identify patients most likely to benefit from vaccination and guide treatment decisions. However, Mauro et al. [37] reported that patients with chronic lymphocytic leukemia previously treated with chemoimmunotherapy did not respond to pneumococcal vaccination. Moreover, age ≥ 60 years, IgG levels < 400 mg/L, prior treatment, and signs of disease progression were associated with a lower response rate in these patients [38].

Evaluation of the cost-effectiveness of PCV vaccination in patients with lung cancer should also be considered. Considering the economic impact of vaccination is critical to understanding its benefits and justifying its inclusion in routine clinical practice. Promoting awareness among healthcare professionals and patients of the importance of PCV vaccination in NSCLC turns out crucial. Increasing knowledge and understanding could lead to higher vaccination rates in this vulnerable population.

This may require long-term observational studies or randomized controlled trials comparing outcomes in vaccinated and unvaccinated NSCLC patients. The results by Mustafa et al. [39] showed that vaccination with PCV13 of patients with multiple myeloma produces a similar immunologic response as compared to normal controls, but the duration of response to vaccination may wane at 180 days compared to the control group. In this study, the response of NSCLC patients with fatal outcomes was statistically lower from the very beginning (7 days after vaccination).

Despite the above conclusions from our study, there are some limitations, such as the fact that due to the diversity of NSCLC and the different therapies used in patients, the correlation between survival and vaccine uptake may be limited to some extent. However, when designing our study, we tried to match patients by gender, age, vaccination status, and smoking status to mitigate these confounding factors as much as possible. Another issue related to the above limitation is heterogeneity in terms of disease stage—this variability is significant because different stages of NSCLC may respond differently to both vaccination and disease progression. Therefore, we performed a statistical analysis comparing the response between early-stage (0–II) and advanced-stage (III–IV) NSCLC groups to address this issue. However, it is important to remember that heterogeneity within these groups may still influence the observed correlation between survival and vaccine uptake. Therefore, further research in this direction is necessary.

These limitations highlight the complexity of interpreting the impact of pneumococcal vaccination on the survival of patients with NSCLC, given the variability in disease progression, response to treatment, and immune system interactions. Despite these challenges, we believe that our findings provide valuable information about the potential benefits of pneumococcal vaccination in patients with NSCLC, warranting further research.

## 5. Conclusions

In this study, we analyzed the comprehensive immunological response in the case of NSCLC patients after anti-pneumococcal vaccination in a 5-year follow-up period. It seemed that the PCV13 vaccination did not increase the overall survival rate in NSCLC patients in this period, and we observed a lack of vaccine response in patients with fatal outcomes, which was definitively correlated with the advanced stage of disease at the time of recruitment. This finding will be elaborated upon much more deeply by our team. The recognized factors associated with survival state were higher anti-pneumococcal IgG concentration and less progressive disease. We hope that the presented long-term research will increase patients′ and physicians′ awareness of the importance of including pneumococcal vaccinations in the standard oncological treatment, which will extend the survival time of lung cancer patients. However, we are aware that NSCLC patients may, therefore, remain at risk of pneumococcal infection despite vaccination, and it remains to be alert about additional strategies to prevent infectious complications in this high-risk population. First, there seems to be a need for more detailed studies to determine whether PCV vaccination positively affects NSCLC progression and treatment response.

## Figures and Tables

**Table 1:** Characteristics of patients from the control and study groups.

	Research Group (*n* = 288)	Control Group (*n* = 69)
Age	Mean ± SD 52.36 ± 9.12 Median (Range) 54.24 (45.00–84.00)	Mean ± SD 53.47 ± 10.14 Median (Range) 55.12 (43.00–79.00)
Sex	156 (54.16%) men 132 (45.8%) women	36 (52.17%) men 33 (47.8) women
Other vaccinations	18.99% of people were vaccinated against influenza (53 people)	20% of patients were additionally vaccinated against influenza
Smoking	7.88% of patients were non-smokers 92.11% of patients smoked cigarettes	9% of patients were non-smokers 91% of patients smoked cigarettes
Surgical procedure	12.19% of patients underwent surgery 87.81% of patients did not undergo surgery	100% of patients did not undergo surgery

**Table 2:** Analysis of selected parameters of peripheral blood count and CRP protein level in NSCLC patients and healthy volunteers before.

Parameters	NSCLC Group (*n* = 288)		Control Group (*n* = 69)		*p*-Value	NSCLC Group (0–II Stages) (*n* = 103)		NSCLC Group (III–IV Stages) (*n* = 182)		*p*-Value
	Mean ± SD	Median (Range)	Mean ± SD	Median (Range)		Mean ± SD	Median (Range)	Mean ± SD	Median (Range)	
WBC [10^3^/mm^3^]	10.10 ± 3.47	9.57 (3.96–30.3)	6.40 ± 1.62	5.95 (3.99–12.63)	<0.0001	9.23 ± 2.5	9.2 (8.7–5.2)	10.63 ± 3.8	9.98 (4.0–30.3)	0.002
LYM [10^3^/mm^3^]	1.86 ± 0.77	1.70 (0.44–4.91)	2.06 ± 0.63	1.94 (0.97–3.76)	0.0055	1.92 ± 0.8	1.68 (0.7–4.9)	1.84 ± 0.7	1.7 (0.4–4.8)	0.71
MON [10^3^/mm^3^]	0.84 ± 0.34	0.76 (0.34–2.5)	0.51 ± 0.15	0.51 (0.24–1.08)	<0.0001	0.81 ± 0.3	0.73 (0.3–2.5)	0.85 ± 0.3	0.8 (0.4–2.0)	0.15
NEU [10^3^/mm^3^]	7.19 ± 3.24	6.57 (1.8–24.94)	3.53 ± 1.21	3.3 (1.63–8.48)	<0.0001	6.53 ± 2.5	6.2 (2.9–15.2)	7.60 ± 3.6	6.9 (1.8–24.9)	0.012
EOS [10^3^/mm^3^]	0.20 ± 0.19	0.15 (0.0–1.5)	0.20 ± 0.15	0.15 (0.0–0.79)	0.99	0.20 ± 0.2	0.15 (0.0–1.1)	0.2 ± 0.19	0.15 (0.0–1.5)	0.99
BAS [10^3^/mm^3^]	0.04 ± 0.03	0.04 (0.0–0.15)	0.04 ± 0.02	0.04 (0.0–0.11)	0.46	0.04 ± 0.03	0.03 (0.01–0.1)	0.04 ± 0.03	0.04 (0.0–0.2)	0.71
RBC [10^6^/mm^3^]	4.55 ± 0.50	4.58 (3.27–6.02)	4.79 ± 0.41	4.83 (3.36–5.5)	<0.0001	4.60 ± 0.5	4.63 (3.3–5.7)	4.52 ± 0.5	4.5 (3.3–6.0)	0.21
HGB [g/dL]	13.26 ± 1.64	13.4 (8.5–17.5)	14.52 ± 1.26	14.6 (11.2–16.8)	<0.0001	13.48 ± 1.5	13.4 (9.3–16.8)	13.12 ± 1.7	13.3 (8.5–17.5)	0.11
HCT [%]	40.30 ± 4.63	40.8 (22.8–56.6)	42.97 ± 3.45	43.3 (31.7–49.9)	<0.0001	40.68 ± 4.8	41.2 (22.8–56.6)	40.04 ± 4.5	40.7 (27.9–56.5)	0.23
PLT [10^3^/mm^3^]	304.72 ± 83.95	307.5 (47.0–529.0)	244.96 ± 58.29	248.0 (117.0–370.0)	<0.0001	300.33 ± 80.2	290.0 (124.0–496.0)	307.68 ± 86.0	312.5 (47.0–529.0)	0.28
CRP [mg/L]	26.77 ± 17.47	23.84 (0.4–89.7)	2.09 ± 2.25	1.26 (0.23–14.4)	<0.0001	25.35 ± 14.8	23.7 (0.9–79.3)	27.75 ± 18.8	24.5 (0.4–89.7)	0.60

Abbreviations: WBC—white blood cells; LYM—lymphocytes; MON—monocytes; NEU—neutrophils; EOS—eosinophils; BAS—basophils; RBC—red blood cells; HGB—hemoglobin; HCT—hematocrit; PLT—pellets; CRP—C-reactive protein.

**Table 3:** Evaluation of anti-pneumococcal IgG antibodies and IgG subclasses in NSCLC patients and controls after PCV13 vaccination at three time points (before vaccination and at the 7^th^ and 30^th^ days after vaccination).

			NSCLC Group (*n* = 288)		Control Group (*n* = 69)		*p*-Value		
			Vaccinated (Group 1) (*n* = 151)	Unvaccinated (Group 2) (*n* = 137)	Vaccinated (Group 3) (*n* = 34)	Unvaccinated (Group 4) (*n* = 35)	1 vs. 2	3 vs. 4	1 vs. 3
Level of IgG1 [g/L]	Before vaccination	Mean ± SD	5.04 ± 1.03	5.21 ± 1.26	6.23 ± 2.06	6.19 ± 1.95	0.069	0.79	0.041
		Median (Range)	4.89 (2.69–8.06)	4.97 (3.02–7.12)	6.22 (3.29–9.14)	6.15 (3.32–8.84)			
	After 7 days	Mean ± SD	5.16 ± 1.47	5.29 ± 1.55	7.33 ± 2.15	6.22 ± 1.73	0.071	0.032	0.0001
		Median (Range)	4.92 (2.79–7.99)	4.83 (2.94–6.83)	7.26 (5.13–10.84)	6.11 (3.12–7.53)			
	After 30 days	Mean ± SD	5.31 ± 1.33	5.37 ± 1.47	7.68 ± 2.34	6.15 ± 1.68	0.97	0.027	0.0001
		Median (Range)	5.02 (3.07–7.57)	4.79 (2.73–6.37)	7.58 (5.73–10.9)	6.21 (3.56–7.62)			
Level of IgG2 [g/L]	Before vaccination	Mean ± SD	1.69 ± 0.63	1.66 ± 0.48	2.04 ± 0.68	2.03 ± 0.64	0.84	0.96	0.032
		Median (Range)	1.63 (0.88–2.65)	1.62 (0.99–2.34)	2.04 (1.08–3.01)	2.02 (1.09–2.92)			
	After 7 days	Mean ± SD	1.78 ± 0.71	1.62 ± 0.51	3.63 ± 0.56	2.08 ± 0.69	0.041	0.0001	0.0001
		Median (Range)	1.86 (0.92–2.73)	1.63 (0.83–2.06)	3.59 (2.17–4.93)	2.05 (1.16–2.84)			
	After 30 days	Mean ± SD	2.68 ± 0.62	1.67 ± 0.49	4.27 ± 0.43	2.18 ± 0.54	0.0001	0.0001	0.0001
		Median (Range)	2.53 (1.31–4.27)	1.65 (0.73–1.85)	4.37 (2.84–5.64)	2.10 (1.34–2.62)			
Level of IgG3 [g/L]	Before vaccination	Mean ± SD	0.21 ± 0.23	0.23 ± 0.11	0.741 ± 0.250	0.73 ± 0.23	0.81	0.83	0.0001
		Median (Range)	0.293 (0.121–0.464)	0.29 (0.11–0.41)	0.74 (0.39–1.10)	0.74 (0.4–1.06)			
	After 7 days	Mean ± SD	0.297 ± 0.183	0.23 ± 0.12	0.78 ± 0.30	0.73 ± 0.22	0.037	0.044	0.00001
		Median (Range)	0.305 (0.136–0.513)	0.26 (0.10–0.39)	0.78 (0.41–1.16)	0.73 (0.40–0.99)			
	After 30 days	Mean ± SD	0.35 ± 0.13	0.23 ± 0.16	0.80 ± 0.14	0.73 ± 0.19	0.01	0.037	0.0001
		Median (Range)	0.35 (0.263–0.615)	0.23 (0.13–0.40)	0.79 (0.44–1.53)	0.73 (0.31–0.84)			
Level of IgG4 [g/L]	Before vaccination	Mean ± SD	0.337 ± 0.215	0.34 ± 0.2	0.38 ± 0.19	0.36 ± 0.29	0.77	0.36	0.087
		Median (Range)	0.29 (0.21–0.53)	0.27 (0.21–0.56)	0.27 (0.25–0.69)	0.27 (0.15–0.4)			
	After 7 days	Mean ± SD	0.35 ± 0.2	0.35 ± 0.17	0.37 ± 0.14	0.36 ± 0.22	0.52	0.44	0. 19
		Median (Range)	0.28 (0.23–0.52)	0.28 (0.21–0.54)	0.27 (0.24–0.67)	0.27 (0.15–0.41)			
	After 30 days	Mean ± SD	0.34 ± 0.17	0.34 ± 0.16	0.37 ± 0.19	0.37 ± 0.18	0.54	0.37	0.82
		Median (Range)	0.29 (0.19–0.48)	0.28 (0.17–0.46)	0.28 (0.20–0.49)	0.27 (0.16–0.43)			

**Table 4:** Evaluation of anti-pneumococcal response in vaccinated NSCLC patients divided regarding the stage of the disease tested before and after 7 and 30 days from the moment of vaccination.

Parameters			NSCLC Group (0–II Stages) (*n* = 103)		NSCLC Group (III–IV Stages) (*n* = 182)		*p*-Value			
			Vaccinated (Group 1) (*n* = 67)	Unvaccinated (Group 2) (*n* = 36)	Vaccinated (Group 3) (*n* = 83)	Unvaccinated (Group 4) (*n* = 99)	1 vs. 2	3 vs. 4	1 vs. 3	2 vs. 4
Level of IgG [g/L]	Before vaccination	Mean ± SD	44.71 ± 11.3	46.07 ± 9.51	47.44 ± 11.8	43.25 ± 13.8	0.4	0.18	0.46	0.21
		Median (Range)	44.6 (16.7–70.0)	47.7 (28.5–59.3)	44.4 (38.1–98.6)	43.8 (12.5–79.7)				
	After 7 days	Mean ± SD	91.38 ± 31.6	51.7 ± 9.6	56.3 ± 13.1	46.7 ± 14.1	<0.0001	<0.0001	<0.0001	0.045
		Median (Range)	92.8 (37.2–156.0)	51.7 (32.4–66.6)	54.6 (42.2–110.9)	47.3 (15.0–83.8)				
	After 30 days	Mean ± SD	407.9 ± 213.6	69.44 ± 15.6	83.4 ± 16.1	51.8 ± 17.8	<0.0001	<0.0001	<0.0001	<0.0001
		Median (Range)	421.5 (60.8–829.6)	72.8 (33.1–90.7)	78.1 (61.0–142.2)	51.1 (15.3–96.0)				
Frequency of total plasmablasts CD19+ in peripheral blood	Before vaccination	Mean ± SD	1.40 ± 1.3	1.06 ± 0.6	1.21 ± 0.93	1.2 ± 0.9	0.55	0.88	0.72	0.56
		Median (Range)	1.0 (0.2–7.3)	0.95 (0.3–2.3)	1.0 (0.1–4.7)	1.0 (0.0–5.4)				
	After 7 days	Mean ± SD	3.13 ± 3.13	1.3 ± 0.74	1.80 ± 0.96	1.32 ± 1.0	<0.0001	0.0001	0.027	0.68
		Median (Range)	2.12 (0.5–17.2)	1.11 (0.4–3.1)	1.5 (0.6–5.4)	1.03 (0.0–5.6)				
	After 30 days	Mean ± SD	4.81 ± 4.9	1.77 ± 1.32	2.23 ± 0.9	1.42 ± 1.3	<0.0001	<0.0001	0.0004	0.17
		Median (Range)	2.9 (0.7–26.9)	1.15 (0.3–4.7)	1.96 (1.1–5.7)	1.10 (0.2–9.6)				
Frequency of IgD-CD19+CD27+++ plasmablasts in CD19+	Before vaccination	Mean ± SD	4.16 ± 5.4	2.9 ± 1.65	3.09 ± 1.88	3.06 ± 2.3	0.67	0.56	0.87	0.81
		Median (Range)	2.76 (0.1–33.1)	2.6 (0.4–7.0)	2.69 (0.1–8.3)	2.59 (0.3–12.1)				
	After 7 days	Mean ± SD	9.02 ± 12.6	3.24 ± 1.8	4.01 ± 1.9	3.31 ± 2.3	<0.0001	0.0034	0.0005	0.68
		Median (Range)	6.04 (0.2–78.0)	2.8 (0.5–7.4)	3.6 (0.8–9.6)	2.8 (0.5–12.4)				
	After 30 days	Mean ± SD	17.38 ± 9.1	1.80 ± 1.5	4.46 ± 1.9	3.12 ± 2.3	<0.0001	<0.0001	<0.0001	0.0002
		Median (Range)	15.5 (1.7–41.3)	1.6 (0.4–7.2)	4.07 (1.2–10.0)	2.59 (0.44–12.3)				
Prevalence of IgD-CD19+CD27+++ plasmablasts in peripheral blood	Before vaccination	Mean ± SD	0.07 ± 0.3	0.02 ± 0.02	0.04 ± 0.04	0.05 ± 0.1	0.96	0.80	0.33	0.38
		Median (Range)	0.02 (0.01–2.4)	0.03 (0.05–0.06)	0.03 (0.01–0.3)	0.03 (0.002–1.0)				
	After 7 days	Mean ± SD	0.16 ± 0.7	0.03 ± 0.02	0.05 ± 0.04	0.05 ± 0.1	<0.0001	0.0008	0.20	0.45
		Median (Range)	0.05 (0.01–5.7)	0.03 (0.01–0.07)	0.04 (0.01–0.25)	0.03 (0.02–1.0)				
	After 30 days	Mean ± SD	0.48 ± 2.2	0.03 ± 0.02	0.06 ± 0.06	0.04 ± 0.04	<0.0001	<0.0001	<0.0001	0.28
		Median (Range)	0.12 (0.03–18.2)	0.03 (0.01–0.07)	0.05 (0.01–0.26)	0.03 (0.0–0.3)				

**Table 5:** Evaluation of the post-vaccination response of NSCLC patients after receiving PCV13 and unvaccinated patients by their survival during the 5 years of follow-up.

Parameters			Dead NSCLC Patients		Alive NSCLC Patients		*p*-Value			
			Vaccinated (Group 1)	Unvaccinated (Group 2)	Vaccinated (Group 3)	Unvaccinated (Group 4)	1 vs. 2	3 vs. 4	1 vs. 3	2 vs. 4
Anti-pneumococcal IgG antibodies [mg/L]	Before vaccination	Mean ± SD	46.89 ± 11.18	43.48 ± 13.87	44.96 ± 12.43	45.12 ± 10.20	0.44	0.94	0.75	0.46
		Median (Range)	44.39 (38.05–98.55)	44.12 (12.54–79.71)	44.56 (16.69–69.97)	44.34 (26.00–64.44)				
	After 1 year	Mean ± SD	81.51 ± 15.13	47.15 ± 14.29	478.16 ± 151.10	75.80 ± 10.16	<0.0001	<0.0001	<0.0001	<0.0001
		Median (Range)	76.72 (59.32–147.22)	48.29 (15.27–85.51)	453.04 (170.09–812.62)	74.38 (54.78–93.21)				
	After 2 years	Mean ± SD	79.47 ± 12.63	45.12 ± 14.25	466.05 ± 147.30	71.15 ± 9.64	<0.0001	<0.0001	<0.0001	<0.0001
		Median (Range)	74.81 (56.88–143.95)	47.25 (14.29–79.69)	449.50 (161.22–794.33)	73.44 (52.49–90.09)				
	After 3 years	Mean ± SD	73.82 ± 5.13	42.36 ± 11.82	415.93 ± 85.21	69.43 ± 9.27	<0.0001	<0.0001	<0.0001	0.047
		Median (Range)	70.19 (55.14–98.24)	43.29 (11.29–75.16)	420.71 (151.36–698.34)	68.89 (50.94–86.37)				
	After 4 years	Mean ± SD	67.80 ± 8.59	42.87 ± 10.55	403.28 ± 71.23	68.37 ± 8.74	<0.0001	<0.0001	<0.0001	0.039
		Median (Range)	62.04 (48.96–81.45)	41.28 (12.36–69.38)	397.69 (136.98–654.71)	67.22 (51.29–85.48)				
	After 5 years	Mean ± SD	54.29 ± 7.13	42.36 ± 9.77	396.58 ± 56.33	66.29 ± 7.26	0.039	<0.0001	<0.0001	0.042
		Median (Range)	52.44 (44.69–75.63)	41.79 (13.26–64.78)	387.15 (132.44–597.36)	66.47 (48.33–77.12)				
Frequency of total plasmablasts CD19+ in peripheral blood [%]	Before vaccination	Mean ± SD	1.20 ± 0.88	1.20 ± 0.89	1.47 ± 1.42	1.11 ± 0.67	0.95	0.74	0.83	0.74
		Median (Range)	1.00 (0.10–4.70)	1.00 (0.05–5.40)	1.00 (0.020–7.30)	1.04 (0.28–2.40)				
	After 1 year	Mean ± SD	2.21 ± 0.9	1.33 ± 1.28	5.42 ± 5.24	1.88 ± 1.20	<0.0001	<0.0001	<0.0001	0.036
		Median (Range)	1.95 (1.10–5.74)	0.90 (0.20–9.60)	3.69 (0.74–26.94)	1.70 (0.20–4.60)				
	After 2 years	Mean ± SD	2.03 ± 0.99	1.24 ± 0.91	5.28 ± 5.11	1.75 ± 0.63	<0.0001	0.001	0.001	0.001
		Median (Range)	1.91 (1.45–3.68)	1.30 (0.39–2.20)	5.09 (1.83–9.00)	1.81 (1.29–2.22)				
	After 3 years	Mean ± SD	1.89 ± 0.40	1.17 ± 0.76	4.71 ± 2.95	1.71 ± 0.61	<0.0001	<0.0001	<0.0001	0.047
		Median (Range)	1.79 (1.41–2.51)	1.19 (0.31–2.07)	4.77 (1.71–7.91)	1.69 (1.25–2.12)				
	After 4 years	Mean ± SD	1.73 ± 0.68	1.18 ± 0.67	4.57 ± 2.47	1.68 ± 0.57	<0.0001	<0.0001	<0.0001	0.037
		Median (Range)	1.58 (1.25–2.08)	1.14 (0.34–1.91)	4.51 (1.55–7.42)	1.65 (1.26–2.10)				
	After 5 years	Mean ± SD	1.39 ± 0.56	1.17 ± 0.63	4.49 ± 1.95	1.63 ± 0.48	<0.0001	<0.0001	<0.0001	0.034
		Median (Range)	1.34 (1.14–1.93)	1.15 (0.36–1.79)	4.39 (1.50–6.77)	1.63 (1.19–1.89)				
Frequency of IgD-CD19+CD27+++ plasmablasts in CD19+ [%]	Before vaccination	Mean ± SD	3.56 ± 3.85	3.01 ± 2.14	2.19 ± 1.24	2.25 ± 1.15	0.43	0.85	0.021	0.072
		Median (Range)	2.69 (0.10–33.07)	2.59 (0.26–12.13)	2.20 (0.11–6.39)	2.23 (0.57–6.39)				
	After 1 year	Mean ± SD	10.37 ± 7.12	3.26 ± 1.93	24.15 ± 9.19	3.77 ± 1.07	0.0001	0.0001	0.0001	0.16
		Median (Range)	7.83 (0.32–26.33)	3.34 (1.06–5.92)	24.26 (1.21- 45.46)	3.71 (2.73–4.65)				
	After 2 years	Mean ± SD	6.19 ± 5.21	3.12 ± 2.20	22.70 ± 14.69	3.56 ± 1.09	0.0001	0.0001	0.0001	0.059
		Median (Range)	5.82 (4.50–11.18)	3.27 (0.98–5.52)	21.89 (7.85–37.81)	3.66 (2.61–4.49)				
	After 3 years	Mean ± SD	5.60 ± 1.63	2.93 ± 1.82	20.26 ± 8.50	3.46 ± 1.04	0.0001	0.0001	0.0001	0.073
		Median (Range)	5.33 (4.18–7.46)	2.99 (0.78–5.21)	20.49 (7.37–34.02)	3.43 (2.43–4.31)				
	After 4 years	Mean ± SD	5.14 ± 1.95	2.57 ± 1.27	19.64 ± 7.10	3.26 ± 0.98	0.0001	0.0001	0.0001	0.031
		Median (Range)	4.71 (2.72–5.18)	2.45 (0.86–4.80)	19.37 (6.67–31.89)	3.21 (2.44–4.26)				
	After 5 years	Mean ± SD	4.12 ± 2.45	2.33 ± 0.51	19.32 ± 5.62	3.30 ± 0.82	0.0001	0.0001	0.0001	0.059
		Median (Range)	3.98 (3.39–5.74)	2.27 (0.91–4.48)	18.86 (6.45–29.09)	3.31 (2.41–3.84)				

**Table 6:** Multivariate analyses of the association between various clinical characteristics and overall survival stage before vaccination and at the 7th and 30th days after vaccination.

Period	Parameters	Wald Stat.	Odds Ratio (95%CI)	*p*-Value
Before vaccination	Intercept	6.42	19.99 (1.96–204.29)	0.012
	Lymphocyte	4.38	1.45 (1.03–2.03)	0.031
	NSCLC stage	18.17	0.96 (0.94–0.98)	<0.0001
7 days after vaccination	Intercept	16.25	0.0006 (<0.0001–0.028)	0.0001
	Platelet	10.63	1.01 (1.003–1.01)	0.0011
	NSCLC stage	8.15	0.97 (0.95–0.99)	0.0043
	ELISA IgG [mg/L]	16.63	1.04 (1.02–1.06)	<0.0001
	Lymphocyte	2.78	1.42 (0.94–2.14)	0.095
	Hemoglobin	16.61	1.46 (1.22–1.74)	<0.0001
30 days after vaccination	Intercept	42.00	<0.0001	<0.0001
	Lymphocyte	4.78	0.42 (0.20–0.92)	0.029
	ELISA IgG [mg/L]	9.12	1.02 (1.01–1.04)	0.003
	Monocytes	10.26	0.12 (0.032–0.43)	<0.0001
	Platelet	4.05	1.01 (1.00–1.013)	0.044
	Hemoglobin	28.66	2.53 (1.80–3.55)	<0.0001
	Leukocytes	14.37	1.36 (1.16–1.59)	<0.0001

**Table 7:** Correlation analysis of the post-vaccination response of NSCLC patients about the tumor stage at the 7th and 30th days after vaccination.

Period	Tumor Stage vs. Parameter	Spearman R	*p*-Value
Before vaccination	Anti-pneumococcal IgG antibodies [mg/L]	−0.035	0.56
	Level of IgG2 [g/L]	−0.035	0.55
	Level of IgG3[g/L]	−0.034	0.56
	Frequency of total plasmablasts CD19+ in peripheral blood [%]	−0.026	0.66
	Frequency of IgD-CD19+CD27+++ plasmablasts in CD19+ [%]	0.031	0.61
	Prevalence of IgD-CD19+CD27+++ plasmablasts in peripheral blood [%]	0.082	0.17
7 days after vaccination	Anti-pneumococcal IgG antibodies [mg/L]	−0.37	<0.0001
	Level of IgG2 [g/L]	−0.19	0.0013
	Level of IgG3 [g/L]	−0.20	0.0018
	Frequency of total plasmablasts CD19+ in peripheral blood [%]	−0.17	0.004
	Frequency of IgD-CD19+CD27+++ plasmablasts in CD19+ [%]	−0.13	0.033
	Prevalence of IgD-CD19+CD27+++ plasmablasts in peripheral blood [%]	−0.038	0.517
30 days after vaccination	Anti-pneumococcal IgG antibodies [mg/L]	−0.45	<0.0001
	Level of IgG2 [g/L]	−0.37	<0.0001
	Level of IgG3 [g/L]	−0.41	<0.0001
	Frequency of total plasmablasts CD19+ in peripheral blood [%]	−0.26	0.0001
	Frequency of IgD-CD19+CD27+++ plasmablasts in CD19+ [%]	−0.25	0.0001
	Prevalence of IgD-CD19+CD27+++ plasmablasts in peripheral blood [%]	−0.33	0.0001

## Data Availability

The data presented in this study are available upon request from the first author.

## Supplementary Materials

The following supporting information can be downloaded at https://www.mdpi.com/article/10.3390/jcm13051520/s1, Table S1 Analysis of selected parameters of peripheral blood and CRP levels in NSCLC patients and healthy volunteers 7 and 30 days after receiving the PCV13 vaccine in relation to unvaccinated patients and Table S2 Analysis of selected parameters of peripheral blood and CRP levels in NSCLC patients divided regarding stage of the disease in 7 and 30 days after receiving the PCV13 vaccine in relation to unvaccinated patients.
