# Supplementary material for: Enhancing Immune Response in Non-Small-Cell Lung Cancer Patients: Impact of the 13-Valent Pneumococcal Conjugate Vaccine
Source: J Clin Med. 2024 Mar 6;13(5):1520. doi: 10.3390/jcm13051520 (PMC10933946; doi:10.3390/jcm13051520)
Supplement: Supplementary file 1 [file jcm-13-01520-s001.zip › jcm-2858243-supplementary.pdf]

Table S1. Analysis of selected parameters of peripheral blood and CRP levels in NSCLC patients and healthy volunteers 7 and 30 days after receiving the PCV13 vaccine in relation to unvaccinated patients

			NSCLC group (n=288)		Control group (n=69)		p-value			
			**Vaccinated (group 1)** (n=151)	**Unvaccinated (group 2)** (n=137)	**Vaccinated (group 3)** (n=34)	**Unvaccinated (group 4)** (n=35)	**1 vs. 2**	**3 vs. 4**	**1 vs. 3**	**2 vs. 4**
WBC $[10^3/mm^3]$	After 7 days	Mean±SD	9.88±2.78	9.73±2.88	6.75±1.79	6.49±1.45	0.52	0.59	<0.0001	<0.0001
		Median (Range)	9.5 (3.91-22.07)	9.25 (4.39-17.91)	6.2 (4.26-12.9)	6.17 (3.64-9.36)				
	After 30 days	Mean±SD	8.56±3.36	9.28±2.98	6.62±1.79	6.73±1.45	0.31	0.63	<0.0001	<0.0001
		Median (Range)	8.75 (0.22-22.19)	8.77 (3.67-17.46)	6.07 (4.13-12.77)	6.44 (3.84-9.56)				
LYM $[10^3/mm^3]$	After 7 days	Mean±SD	1.91±0.77	1.74±0.82	2.28±0.6	2.25±0.65	0.047	0.68	0.0007	0.0001
		Median (Range)	1.73 (0.6-4.99)	1.63 (0.2-4.58)	2.11 (1.26-3.67)	2.11 (1.31-3.88)				
	After 30 days	Mean±SD	1.93±0.79	1.30±0.67	2.19±0.61	2.37±0.66	<0.0001	0.33	0.012	<0.0001
		Median (Range)	1.76 (0.55-5.21)	1.17 (0.11-3.11)	2.03 (1.17-3.52)	2.23 (1.41-4.05)				
MON $[10^3/mm^3]$	After 7 days	Mean±SD	0.76±0.32	0.79±0.35	0.50±0.14	0.48±0.16	0.47	0.33	<0.0001	<0.0001
		Median (Range)	0.72 (0.22-1.64)	0.75 (0.22-2.62)	0.52 (0.25-0.72)	0.46 (0.22-1.06)				
	After 30 days	Mean±SD	0.70±0.39	0.69±0.34	0.53±0.14	0.41±0.16	0.56	0.0008	0.097	<0.0001
		Median (Range)	0.59 (0.07-2.08)	0.66 (0.08-2.39)	0.55 (0.28-0.75)	0.39 (0.15-0.99)				
NEU $[10^3/mm^3]$	After 7 days	Mean±SD	6.41±2.43	6.69±3.64	3.72±1.34	3.65±1.09	0.73	0.87	<0.0001	<0.0001
		Median (Range)	6.08 (1.09-14.11)	5.64 (1.44-23.8)	3.58 (1.7-8.55)	3.51 (1.98-7.23)				
	After 30 days	Mean±SD	6.28±3.33	5.74±2.68	3.56±1.37	3.76±1.09	0.90	0.56	<0.0001	<0.0001
		Median (Range)	5.66 (0.35-21.4)	5.15 (0.91-16.1)	3.53 (1.37-8.22)	3.62 (2.09-7.34)				
EOS $[10^3/mm^3]$	After 7 days	Mean±SD	0.20±0.17	0.19±0.15	0.23±0.15	0.23±0.16	0.92	0.89	0.096	0.12
		Median (Range)	0.16 (0.01-1.02)	0.16 (0.0-0.87)	0.17 (0.05-0.77)	0.19 (0.03-0.82)				
	After 30 days	Mean±SD	0.16±0.2	0.90±8.6	0.21±0.15	0.29±0.16	0.67	0.0052	0.0034	<0.0001
		Median (Range)	0.10 (0.0-1.89)	0.13 (0.0-101.0)	0.17 (0.04-0.73)	0.25 (0.09-0.88)				
RBC $[10^6/mm^3]$	After 7 days	Mean±SD	4.86±0.49	4.45±0.58	4.63±0.41	4.9±0.42	<0.0001	0.0058	0.008	<0.0001
		Median (Range)	4.83 (3.56-6.26)	4.49 (3.1-5.93)	4.67 (3.19-5.29)	4.92 (3.98-5.62)				
	After 30 days	Mean±SD	4.30±0.47	4.31±0.59	4.72±0.41	5.05±0.42	0.85	0.0014	<0.0001	<0.0001
		Median (Range)	4.28 (3.09-5.79)	4.33 (3.09-5.67)	4.76 (3.28-5.38)	5.06 (4.12-5.76)				
HGB [g/dL]	After 7 days	Mean±SD	13.93±1.63	11.55±2.27	14.37±1.27	14.28±1.26	<0.0001	0.76	0.11	<0.0001
		Median (Range)	14.13 (9.7-17.93)	11.51 (5.31-17.2)	14.44 (11.04-16.54)	14.36 (11.76-16.56)				
	After 30 days	Mean±SD	11.42±1.83	10.24±2.29	14.49±1.13	14.4±1.26	<0.0001	0.70	<0.0001	<0.0001
		Median (Range)	11.65 (6.35-15.35)	9.99 (5.14-16.39)	14.6 (11.35-16.2)	14.48 (11.88-16.68)				
HCT [%]	After 7 days	Mean±SD	40.69±4.02	38.29±5.47	42.89±3.77	40.43±3.16	<0.0001	0.0034	0.0037	0.0099
		Median (Range)	41.01 (29.61-50.15)	38.59 (23.34-57.14)	43.13 (31.53-49.73)	40.95 (33.15-44.95)				
		Mean±SD	36.87±4.85	37.12±6.13	42.97±3.44	39.07±3.16	0.56	<0.0001	<0.0001	0.089

	After 30 days	Median (Range)	37.85 (22.34-46.34)	37.3 (20.19-56.11)	43.6 (32.9-48.4)	39.59 (31.79-43.59)				
CRP [mg/L]	After 7 days	Mean±SD	26.72±14.16	32.71±18.02	2.74±1.94	1.97±2.51	0.006	0.014	<0.0001	<0.0001
		Median (Range)	26.15 (0.2-76.95)	31.62 (4.34-90.6)	2.31 (0.62-8.32)	1.08 (0.73-14.53)				
	After 30 days	Mean±SD	24.89±16.53	48.14±22.56	2.50±1.94	2.03±2.51	<0.0001	0.13	<0.0001	<0.0001
		Median (Range)	21.9 (0.84-84.22)	44.74 (7.06-114.79)	2.07 (0.38-8.08)	1.14 (0.74-14.63)				

* statistically significant result

Table S2. Analysis of selected parameters of peripheral blood and CRP levels in NSCLC patients divided regarding stage of the disease in 7 and 30 days after receiving the PCV13 vaccine in relation to unvaccinated patients.

			NSCLC group (0-II stages) (n=103)		NSCLC group (III-IV stages) (n=182)		p-value			
			**Vaccinated (group 1)** (n=67)	**Unvaccinated (group 2)** (n=36)	**Vaccinated (group 3)** (n=83)	**Unvaccinated (group 4)** (n=99)	**1 vs. 2**	**3 vs. 4**	**1 vs. 3**	**2 vs. 4**
WBC [10^3/mm^3]	After 7 days	Mean±SD	9.60±2.64	8.98±2.88	10.11±2.9	10.06±3.05	0.2	0.71	0.21	0.063
		Median (Range)	9.0 (5.4-22.07)	9.25 (4.39-17.91)	10.1 (3.9-16.2)	9.5 (4.4-17.9)				
	After 30 days	Mean±SD	9.5±2.8	8.85±2.17	8.2±3.7	9.49±3.20	0.23	0.02	0.026	0.41
		Median (Range)	9.02 (4.3-22.19)	8.69 (4.23-16.44)	8.2 (0.2-16.7)	9.1 (3.7-17.5)				
LYM [10^3/mm^3]	After 7 days	Mean±SD	1.94±0.78	1.96±0.92	1.88±0.8	1.67±0.77	0.64	0.09	0.68	0.21
		Median (Range)	1.70 (0.9-4.99)	1.50 (0.45-3.87)	1.7 (0.6-4.7)	1.6 (0.2-4.6)				
	After 30 days	Mean±SD	2.1±0.8	1.39±0.69	1.8±0.8	1.27±0.66	<0.0001	<0.0001	0.007	0.56
		Median (Range)	1.86 (1.1-5.21)	1.07 (0.11-2.63)	1.6 (0.6-4.75)	1.2 (0.2-3.1)				
MON [10^3/mm^3]	After 7 days	Mean±SD	0.69±0.3	0.77±0.49	0.82±0.33	0.80±0.29	0.57	0.90	0.012	0.075
		Median (Range)	0.64 (0.22-1.62)	0.65 (0.22-2.62)	0.78 (0.3-1.6)	0.77 (0.3-1.9)				
	After 30 days	Mean±SD	0.55±0.3	0.60±0.49	0.82±0.42	0.72±0.26	0.91	0.40	<0.0001	0.0004
		Median (Range)	0.50 (0.07-1.5)	0.53 (0.09-2.39)	0.72 (0.08-2.1)	0.73 (0.1-1.3)				
NEU [10^3/mm^3]	After 7 days	Mean±SD	6.18±2.2	6.58±3.2	6.62±2.6	6.79±3.8	0.83	0.47	0.33	0.88
		Median (Range)	5.85 (2.62-14.11)	5.38 (2.34-16.11)	6.2 (1.1-12.3)	5.97 (1.4-23.8)				
	After 30 days	Mean±SD	5.72±2.4	5.99±2.77	6.75±3.9	5.70±2.7	0.69	0.17	0.28	0.65
		Median (Range)	5.6 (1.82-13.9)	5.15 (1.81-15.8)	5.73 (0.35-21.4)	5.2 (0.9-16.1)				
		Mean±SD	0.20±0.19	0.16±0.09	0.20±0.16	0.20±0.17	0.99	0.77	0.47	0.52

EOS [10^3/mm^3]	After 7 days	Median (Range)	0.15 (0.01-1.02)	0.15 (0.02-0.37)	0.16 (0.03-0.71)	0.17 (0.0-0.9)0.93				
	After 30 days	Mean±SD	0.18±0.26	0.19±0.23	0.15±0.13	1.17±10.1	0.11	0.72	0.93	0.068
		Median (Range)	0.10 (0.0-1.89)	0.14 (0.03-1.42)	0.11 (0.0-0.56)	0.1 (0.0-101.0)				
RBC [10^6/mm^3]	After 7 days	Mean±SD	4.90±0.48	4.69±0.5	4.82±0.5	4.36±0.58	0.04	<0.0001	0.35	0.002
		Median (Range)	4.93 (3.56-5.96)	4.71 (3.8-5.76)	4.78 (3.6-6.26)	4.4 (3.1-5.9)				
	After 30 days	Mean±SD	4.37±0.41	4.53±0.50	4.24±0.51	4.23±0.61	0.07	0.84	0.084	0.007
		Median (Range)	4.37 (3.45-5.32)	4.62 (3.6-5.5)	4.23 (3.1-5.8)	4.2 (3.1-5.7)				
HGB [g/dL]	After 7 days	Mean±SD	14.51±1.42	12.86±1.9	13.48±1.7	11.05±2.21	<0.0001	<0.0001	<0.0001	<0.0001
		Median (Range)	14.43 (10.43-17.93)	12.8 (8.9-15.9)	13.7 (9.7-17.7)	11.1 (5.3-17.2)				
	After 30 days	Mean±SD	12.41±1.4	11.68±2.2	10.65±1.8	9.71±2.11	0.11	0.0001	<0.0001	<0.0001
		Median (Range)	12.5 (8.25-15.2)	11.6 (6.8-15.1)	10.9 (6.4-15.4)	9.6 (5.1-16.4)				
HCT [%]	After 7 days	Mean±SD	41.28±3.9	39.6±6.0	40.25±4.1	37.73±5.22	0.13	<0.0001	0.23	0.056
		Median (Range)	41.45 (30.15-50.15)	39.99 (23.34-57.14)	40.8 (29.6-47.6)	38.1 (24.8-57.0)				
	After 30 days	Mean±SD	38.18±3.96	39.4±6.1	35.8±5.3	36.30±6.02	0.075	0.58	0.006	0.003
		Median (Range)	38.6 (25.47-44.4)	40.2 (22.3-56.11)	36.3 (22.3-46.3)	36.9 (20.2-56.0)				
CRP [mg/L]	After 7 days	Mean±SD	24.0±15.8	27.4±13.4	29.10±12.24	34.77±19.2	0.15	0.09	0.006	0.12
		Median (Range)	21.77 (0.2-76.95)	26.1 (4.9-52.0)	26.7 (7.0-67.3)	31.9 (4.3-90.6)				
	After 30 days	Mean±SD	19.2±15.3	35.2±16.65	29.45±16.3	52.82±22.85	<0.0001	<0.0001	<0.0001	<0.0001
		Median (Range)	17.0 (0.84-72.7)	33.7 (7.06-74.7)	25.7 (9.5-84.2)	50.4 (12.7-114.8)				
